# The impact of a psychiatry clinical rotation on the attitude of South African final year medical students towards mental illness

**DOI:** 10.1186/s12909-019-1543-9

**Published:** 2019-04-25

**Authors:** Caro De Witt, Inge Smit, Esmè Jordaan, Liezl Koen, Dana J. H. Niehaus, Ulla Botha

**Affiliations:** 1 Department of Psychiatry, Faculty of Medicine and Health Sciences, Stellenbosch University, PO Box 19063, Cape Town, Tygerberg 7505 South Africa; 2 Biostatistics Unit, Medical Research Council, Parow, South Africa and Statistics and Population Studies Department, University of the Western Cape, Cape Town, South Africa

**Keywords:** Stigma, Mental illness, Medical students, Clinical training

## Abstract

**Background:**

Stigmatising attitudes of health care professionals towards mental illness can impede treatment provided for psychiatric patients. Many studies have reported undergraduate training to be a critical period for changing the attitudes of medical students, and one particularly valuable intervention strategy involves time spent in a clinical psychiatric rotation. In South Africa, medical students are exposed to a clinical rotation in psychiatry but there is no evidence to show whether this has an effect on attitudes toward mental illness.

**Methods:**

This prospective cohort study involved a convenience sample of 112 South African medical students in their 5th or 6th year of undergraduate training. This sample attended a 7-week psychiatry rotation. The Attitudes to Mental Illness Questionnaire (AMIQ) was used to assess students’ attitudes toward mental illness before and after the clinical rotation which includes exposure to a number of psychiatric sub-divisions and limited didactic inputs.

**Results:**

There was a significant improvement (*p* < 0.01, *t*-test) in the students’ attitude toward mental illness following the psychiatric rotation. Females displayed a more positive attitude towards mental illness at the end of the rotation compared to males. The participants’ attitude significantly deteriorated for the non-psychiatric vignette describing diabetes (< 0.01, *t*-test).

**Conclusions:**

Our findings suggest that clinical training and exposure to a psychiatric setting impacts positively on medical students’ attitude towards mental illness, even when this training does not include any focused, didactic anti-stigma input.

## Background

Stigmatising attitudes of health care professionals towards mental illness can impede treatment provided for patients suffering from psychiatric problems [1]. The attitudes of medical students toward mental illness is an important concern owing to the fact that these individuals are likely to encounter and care for mentally-ill patients, either directly or indirectly, as future doctors [2]. Their attitude will affect the quality of service they provide to these patients and their families and may influence how individuals seek professional help for psychiatric problems [3]. The attitudes of health care professionals can also have implications for national mental health policies and programs, which in turn, impact on the quality of care and investment in mental health care facilities [4]. Negative attitudes might also mitigate against psychiatry as a future choice of specialization for medical students [5], which is important considering the critical shortage of psychiatrists, both locally and abroad [6, 7].

Several studies have examined stigmatising attitudes towards mental illness amongst students from both developed and developing countries [5, 8–11], but South African students have received limited attention. The general South African public is known to have stigmatizing attitudes toward mentally-ill people, especially toward individuals with substance use disorders and schizophrenia [12]. Furthermore, South Africa’s significant cultural and ethnic diversity may impact the way these attitudes manifest in our society [13]. Local studies have shown that many African individuals perceive mental illness to be a form of bewitchment or possession by evil spirits [14, 15]. Mbanga showed that many Xhosa family members of schizophrenia patients believed those affected were “dirty”, “weak”, “unpredictable” and “dangerous” [15]. It is likely that our South African medical students might share some of the beliefs and negative attitudes that are characteristic of our local setting.

A number of studies have reported undergraduate training to be a critical period for changing the attitudes of medical students toward mental illness [16, 17]. Students’ attitudes and opinions appear to be more responsive to change during the early years of medical education [18], which means that the undergraduate years might be a critical time to introduce certain intervention strategies. One strategy, which has consistently been shown to improve students’ attitude towards mental illness, involves time spent in a clinical psychiatric rotation [5]. Studies performed in Europe, America, Canada and Australia have shown that medical students’ attitude toward mental illness is more positive following a clinical rotation [19–23]; but there is far less data available from middle- and low-income countries (LMICs). Studies performed in Malaysia and Nigeria showed improvement in female students’ attitude towards mental illness after a clinical rotation in psychiatry, but not in males [11, 24]. In South Africa, medical students are exposed to a clinical rotation in psychiatry but there is no evidence to show whether this has an effect on attitudes toward mental illness. Therefore, this study aimed to assess medical students’ attitudes toward mental illness, and whether these attitudes changed after a clinical rotation in psychiatry.

## Methods

### Study design, setting and participants

This pre- and post-design study was conducted in 2016 at Stellenbosch University, South Africa. We used a convenience sample of 112 medical students in their 5th or 6th year of undergraduate training. This sample attended a 7-week psychiatry rotation.

### Description of psychiatry rotation

The psychiatry rotation at Stellenbosch University consists of seven weeks of clinical training and exposure to several different clinical units. Two-thirds of the rotation is spent at Stikland Psychiatric Hospital where students divide their time evenly between acute adult services, and one of the specialized units. Students are randomly allocated to the specialized units, which include addiction services (alcohol rehabilitation and opioid detoxification), therapeutic services or old age psychiatry. The remaining third of the rotation is spent at a child and adolescent psychiatry unit, located at either Lentegeur Psychiatric Hospital or the Tygerberg Academic Complex. During the 7-week rotation, the vast majority of time is spent in a clinical setting where students form part of the clinical teams, attend ward rounds and conduct comprehensive assessments on both in- and out-patients. All clinical exposure is supervised by psychiatric registrars or qualified psychiatrists. Additionally, the students are exposed to a small number of formal educational interventions, such as focused tutorials on specific topics and video-based assessments. None of these interventions specifically focuses on stigma.

### Data collection

The students met with the researcher on the first and last day of their clinical rotation and completed the Attitudes to Mental Illness Questionnaire (AMIQ) [25]. On the first day, the students were unaware that the AMIQ would be repeated at the end of the rotation. The participants rotated through different specialized units based on a random allocation roster drawn up by the rotation chair. Of 112 medical students, 39 were allocated to old age psychiatry, 28 to addiction services, and 45 to therapeutic services. The pre- and post-intervention AMIQ assessments were completed by 112 and 107, participants respectively. Two students did not complete the post-intervention assessment due to absence on the day, and an additional three post-intervention assessments were incomplete. Code numbers were assigned to each participant to allow for paired t-testing during analysis.

### Measures

The AMIQ is a brief, self-administered questionnaire that has been shown to have good psychometric properties [25]. The questionnaire consists of seven case vignettes, four of which refer to psychiatric cases, while the remaining three refer to non-psychiatric cases. Each vignette has five questions, which explore the participants’ attitudes towards the hypothetic patient. Responses are scored on a 5-point Likert scale (maximum 2, minimum − 2, with ‘neutral’ and ‘don’t know’ being scored 0). The score for each question is summed to give a total score between − 10 and 10. Lower scores indicate more negative attitudes.

### Statistical analyses

Continuous variables were summarized as means with standard deviation, while categorical variables were summarized as counts and percentages. Paired t-tests were used to assess the change in the mean combined score for the psychiatric cases, as well as the change in scores for each individual vignette. *t*-tests were also used to assess the relationship between gender/specialist rotation and attitude scores, while the Pearson product-moment correlation coefficient was used to assess the relationship between age and attitude. Statistical significance was evaluated at the 5% significance level (*p* < 0.05) and all analyses were performed using IBM SPSS Statistics for Windows, version 24 (IBM Corp., Armonk, N.Y., USA).

### Ethical considerations

Study approval was granted by the Health Research Ethics Committee of Stellenbosch University (study number N08/04/097), as well as the Tygerberg Facility Management and Ethics Committee. The study was conducted in accordance with the South African  Good Clinical Practice Guidelines (DOH 2006), and the Declaration of Helsinki (2013). Participation was voluntary and all participants provided written, informed consent. The survey questionnaire was administered in an anonymous form and no identifiable information was collected from the participants.

## Results

More than half (56%) of all participants (*N* = 112) were female, and the mean age (±SD) was 24.1 (± 2.1) years. The participant age distribution was skewed with a cumulative percentage of 95.5% at 27 years (age range 22–38 years). We found that students’ attitude towards mental illness was negative before the clinical rotation, with a mean (±SD) combined score for the psychiatry cases of − 6.86 (±8.30). After completion of the psychiatric rotation, there was an overall improvement in the students’ attitude toward mental illness. In particular, the mean (±SD) combined score for the psychiatric cases significantly increased (t(106) = − 3.041, *p* = 0.003; *t*-test) to − 4.79 (±8.40). Though there was a significant improvement in this score, the students’ attitude remained negative towards the psychiatric cases. Furthermore, individual scores significantly improved for the psychiatric vignettes describing heroin use and depression with overdose, but not for the vignette describing schizophrenia (Table 1). For the non-psychiatric vignettes, the participants’ attitude significantly deteriorated for the vignette describing diabetes. However, the scores were still fairly positive compared to scores for psychiatric cases.

**Table 1:** Differences in mean AMIQ scores among final year medical students before and after a clinical psychiatry rotation

Vignette	Mean scores (±SD)			
	Pre-rotation (*N* = 112)	Post-rotation (*N* = 107)	t	*p*
Psychiatric cases				
Heroin-use	−6.07 (2.67)	−5.11 (2.89)	−3.281	< 0.01*
Depression with overdose	1.10 (2.82)	1.83 (2.71)	−2.665	< 0.01*
Alcohol abuse	0.5 (3.80)	0.48 (3.80)	−0.148	0.88
Patient with schizophrenia	−2.38(3.42)	−1.95 (3.13)	−1.134	0.26
Non-psychiatric cases				
Convicted criminal	−6.69 (2.86)	−6.86 (2.29)	0.317	0.75
Patient with diabetes	7.63 (2.54)	6.67 (2.64)	3.679	< 0.01*
Churchgoing Christian	6.21 (3.06)	6.03 (2.97)	00.840	0.40

*indicates statistically significant *t*-test at *p* < 0.05

There was no significant difference (t(110) = −0.841, *p* = 0.40; *t*-test) in pre-rotation scores for male (mean score for combined psychiatric cases ±SD, −7.59 ± 9.07) and female students (mean ± SD, − 6.27 ± 7.55). Female students had a more positive attitude towards mental illness (mean score for combined psychiatric cases ±SD, − 3.42 ± 7.52) at the end of the rotation compared to the male students (mean ± SD, − 6.55 ± 9.19), but this difference was not statistically significant (t(110) = − 0.841, *p* = 0.06; *t*-test). In terms of individual vignettes, the attitude of males (mean ± SD, − 5.88 ± 2.52) toward heroin use was significantly more negative (t(108) = − 2.504, *p* = 0.01; *t*-test) compared to females (mean ± SD, − 4.52 ± 3.03). Males also viewed schizophrenia more negatively (mean ± SD, − 2.72 ± 3.18) compared to females (mean ± SD, − 1.35 ± 2.98; t(105) = − 2.297, *p* = 0.02; *t*-test). Age did not have a significant impact on the post-rotation attitudes of participants (Pearson product-moment correlation coefficient, r = 0.07, *p* = 0.45). Rotation through different specialized units at Stikland Hospital (addictions, therapeutic, old age) did not affect students’ attitudes to the psychiatric and non-psychiatric vignettes (*p* > 0.05; *t*-test).

## Discussion

This study assessed the impact of a seven-week clinical psychiatric rotation on the attitudes of undergraduate medical students towards psychiatric patients and mental illness. Our findings suggest that a clinical rotation in psychiatry, even without specific anti-stigma input, did have a positive influence on South African medical students’ attitude towards mental illness.

The majority of students had a negative attitude towards psychiatry prior to the rotation, which is, perhaps, unsurprising considering the prevalence of stigmatizing attitudes toward mentally-ill patients in the general South African population [12–15]. Our participants showed a significant improvement in their attitude toward mental illness after the rotation, a finding similar to that of other authors [5]. However, the students’ overall attitude towards psychiatry remained negative. Although our findings highlight the positive impact of psychiatry clinical rotations on medical students’ attitude to psychiatry, other studies have shown significantly more negative attitudes after a clinical rotation [10, 21, 26]. These inconsistent effects could be related to certain pedagogical factors that may affect students’ learning experiences [27]. For example, an unfavourable training environment may serve to heighten the negative attitudes of students towards people with mental illness [28]. There may be a need to develop specific pedagogic theory for clinical rotations, and better define key factors that contribute to effective learning and teaching through these strategies.

Female medical students displayed a more positive attitude to mental illness, which is similar to findings in other studies [10, 11, 24, 29]. This might demonstrate that female students are more tolerant of undesirable behaviour caused by substance use or mental illness [24]. Age did not have a significant impact on student attitudes toward mental illness in the current study, but this variable has been shown to influence attitudes in other studies. For example, Abedowale et al. [11] reported that students below the age of 25 had a greater improvement in their attitudes after a clinical rotation, compared to students above age 25. Furthermore, Roman et al. reported a greater improvement in the attitudes of younger students toward mental illness following a psychiatry rotation [30]. Future studies should possibly assess the impact of a clinical rotation at an earlier stage in the undergraduate medical training program.

Assessment after the rotation showed a significant worsening of students’ attitudes towards this diabetes vignette. There tends to be a higher incidence of type II diabetes among psychiatric patients, and one could speculate that clinical rotation in psychiatry highlighted the therapeutic challenges associated with treating mental illness comorbid with diabetes [31–33]. Generally, there appears to be a great dearth of literature concerning students’ attitudes towards diabetes. Our study highlights a need to assess and address negative attitudes towards diabetes, especially in the context of its relationship with psychiatric illness.

One of the major limitations of this study was its relatively small sample size. Furthermore, this study was restricted to a single South African university, which means our results have limited generalizability. Given the demographic diversity of South Africans, future studies should assess ethnic differences in stigmatising attitudes toward people with mental illness. Qualitative approaches would also be helpful to explore factors that contribute to observed changes in students’ attitudes, including the attitudes of the teachers, site of the training and personal history of mental illness. Future studies should also assess the impact of psychiatry clinical rotations on medical students’ attitude to psychiatry as a career, as this might enhance recruitment into the specialty.

## Conclusions

Our findings show that a clinical rotation in psychiatry, offered to 5th and 6th year South African medical students, positively impacts students’ attitudes toward mental illness, even in the absence of focused anti-stigma interventions. However, even though there was an improvement in attitudes after the rotation, those toward mental illness were still negative. Our results contribute to the existing research on medical students and their negative attitude towards mental illness. We emphasise the value of clinical rotations and other necessary strategies aimed at reducing stigmatising attitudes among trainees. Attitudes toward diabetes were found to be positive both before and after the rotation, but deteriorated after the intervention. Further studies are needed to assess and address these attitudes to ensure quality care is provided for patients with mental illness comorbid with diabetes mellitus.

## Abbreviations

AMIQ: Attitudes to Mental Illness Questionnaire
SD: Standard deviation
SD: Standard deviation
WHO: World Health Organization
